# Single-Nucleotide Polymorphisms Related to Glioblastoma Risk and Worldwide Epidemiology: A Systematic Review and Meta-Analysis

**DOI:** 10.3390/jpm15090401

**Published:** 2025-09-01

**Authors:** Giovanna Gilioli da Costa Nunes, Francisco Cezar Aquino de Moraes, Rita de Cássia Calderaro Coelho, Marianne Rodrigues Fernandes, Sidney Emanuel Batista dos Santos, Ney Pereira Carneiro dos Santos

**Affiliations:** 1 Research Center of Oncology, Federal University of Pará Belém, Belém 66073-000, Brazil; francisco.cezar2205@gmail.com (F.C.A.d.M.); rccalderarocoelho@gmail.com (R.d.C.C.C.); fernandesmr@yahoo.com.br (M.R.F.); npcsantos.ufpa@gmail.com (N.P.C.d.S.); 2 Faculty of Medicine, Federal University of Pará Belém, Belém 66073-000, Brazil; 3 Laboratory of Human and Medical Genetics, Institute of Biological Science, Federal University of Pará, Belém 66077-830, Brazil; sidneysantosufpa@gmail.com

**Keywords:** glioblastoma, epidemiology, risk, single-nucleotide polymorphism

## Abstract

**Background/Objectives**: Glioblastomas are a part of adult-type diffuse gliomas, the most common and most aggressive primary brain tumors in adults (glioblastoma, IDH-wildtype). The identification of the genetic factors associated with glioblastoma could be an important contribution to the diagnosis and early prevention of this disease. We compiled data from the global literature and analyzed clinically relevant variants implicated in glioblastoma risk. **Methods**: PubMed, Web of Science, and Scopus were used as databases. Associations between the SNPs and glioblastoma risk were calculated as a measure of pooled odds ratios (ORs) and 95% confidence intervals. Pearson’s analysis was used for epidemiological correlation (only *p*-values less than 0.05 were statistically significant), and data were obtained from the World Health Organization platform and the 1000 Genomes Project. Statistical analysis was performed using Review Manager (RevMan) 5.4 and BioEstat 5.0. **Results**: *CCDC26* rs891835 G/T, G/G, and G/T-G/G genotypes were analyzed and determined to increase glioblastoma risk (G/T OR = 1.96, 95% CI: 1.38–2.77, *p* = 0.0002, I^2^ = 0%; G/G OR = 1.33, 95% CI: 0.46–3.85, *p* = 0.60, I^2^ = 0%; G/T − G/G OR = 1.96, 95% CI: 1.39–2.76, *p* = 0.0001, I^2^ = 0%). Epidemiological correlation also demonstrated that the higher the frequency of the *CCDC26* rs891835 variant, the higher the incidence of that variant in the European population. **Conclusions**: *CCDC26* rs891835 may serve as a predictive biomarker for glioblastoma, IDH-wildtype risk and may influence higher glioblastoma incidence rates in the European population.

## 1. Introduction

Brain tumors represent a diverse group of neoplasms, encompassing over 50 distinct entities. The fifth edition of the WHO Classification of Tumors of the Central Nervous System (WHO CNS5) categorizes gliomas, glioneuronal tumors, and neuronal tumors into six families. Among these, adult-type diffuse gliomas are the most prevalent and aggressive primary brain tumors in adults, with glioblastoma, IDH-wildtype being a notable example. A significant advancement in the latest update of the WHO CNS tumor classification is the incorporation of molecular findings into the diagnostic framework for adult-type diffuse gliomas, moving beyond sole reliance on morphological characteristics and integrating specific genetic modifiers [1].

Glioblastoma, IDH-wildtype (GBM), arises from astrocytic glial cells and represents the highest-grade malignant glioma. The average annual age-adjusted incidence rate for all primary brain tumors is approximately 24.25 per 100,000, with malignant tumors at 6.94 and non-malignant tumors at 17.88 [2]. These tumors are typically associated with a dismal prognosis and poor quality of life. This disease is most common in individuals aged 65 years and older, with factors such as extensive necrosis and MGMT promoter methylation status—primarily linked to predicting therapeutic response—playing significant roles [3].

Previous investigations have demonstrated that factors such as familial cancer history, which accounts for approximately 5% of cases, serve as risk factors for glioma; however, these account for only a minor proportion of glioma cases [4]. The majority of glioma cases occur in individuals without a family history of glioma; a significant genetic component, marked by the co-inheritance of several low-risk genetic variants, also contributes to the susceptibility to glioma [5].

The diagnosis of GBM is particularly challenging due to its frequent intratumoral morphological heterogeneity. Moreover, the brain's susceptibility to damage from conventional therapies makes GBM highly resistant to treatment, often resulting in a poor prognosis for patients. Many systemic cancer drugs are unable to cross the blood–brain barrier, and complete tumor resection during surgery remains difficult [6].

Identifying the genetic factors associated with GBM could significantly contribute to improving diagnosis and enabling early prevention of this disease. In this context, we focus on a comprehensive evaluation of key genes implicated in glioblastoma susceptibility, including *IL-4R*, *CCDC26*, *GSTP1*, *AURKB*, *AURKC*, *STAT5B*, *CHEK2*, *IL-3*, *IL-10*, *PARP1*, *PRKDC*, *MLH1*, *ERCC2*, *FLT3*, *XRCC5*, *NFIL3*, *TP53*, *BRCA1*, and *EGFR.*

The aim of this study is to compile data from the global literature and analyze clinically relevant variants and genes implicated in the heightened risk and increased susceptibility to GBM. Furthermore, the study seeks to establish correlations between these variants and the worldwide epidemiology of brain and central nervous system cancer.

## 2. Materials and Methods

### 2.1. Protocol and Registration

This systematic review followed the Preferred Reporting Items for Systematic Reviews and Meta-Analysis (PRISMA) guidelines [7]. The protocol was registered in the International Prospective Register of Systematic Reviews (PROSPERO) with registration identification CRD42024601104.

### 2.2. Search Strategy 

A comprehensive literature search was performed to identify relevant studies published up to 11 March 2024, using the PubMed, Scopus, and Web of Science databases. The search strategy, including MeSH terms, is detailed in Table S2 of the Supplementary Material. To include additional studies, references from the selected articles and systematic reviews were also examined, and alerts were set up in each database to notify of any newly published studies matching the search criteria. Identified articles were imported into the reference management software (EndNote^®^, version X7, Thomson Reuters, Philadelphia, PA, USA), and duplicates were removed both automatically and manually. Titles and abstracts were independently screened by two reviewers (G.G.d.C.N. and F.C.A.d.M.), with disagreements resolved through consensus between the two reviewers and the senior author (N.P.C.S.).

### 2.3. Study Selection 

The PECOS model (Population, Exposure, Comparator, Outcomes, and Study Design) was applied to select potential studies: P (Population), patients diagnosed with GBM (glioma IV); E (Exposure) and C (Comparator), SNPs associated with GBM risk and different genotypes; O (Outcomes), susceptibility or increased risk of developing GBM; and S (Study Design), observational studies (cohort, case-control, or cross-sectional). Exclusion criteria included conference abstracts, preprints, theses, dissertations, and studies relying on biobanks. Two reviewers (G.G.d.C.N. and F.C.A.d.M.) independently screened the titles and abstracts of the citations to identify potentially relevant studies. Full-text articles were then retrieved and reviewed independently by the same two reviewers based on the inclusion criteria, with a third reviewer (R.d.C.C.C.) resolving any disagreements or uncertainties. The screening process was conducted using Rayyan QCRI, a free web-based tool designed to support researchers in systematic reviews.

### 2.4. Data Extraction 

The following data were extracted by three independent reviewers (G.G.d.C.N, F.C.A.d.M., and R.d.C.C.C.) using standardized sheets in Microsoft Excel, Version 2016: authors; publication year; country where the study was conducted; total number, gender, ethnicity, mean age, and mean age at diagnosis of participants with GBM; genotyping method; histologically confirmed GBM; and the authors’ main findings. Disagreements were resolved through discussion with the senior author (N.P.C.S.).

### 2.5. Quality Assessment 

The Newcastle–Ottawa Scale (NOS) [8] was utilized to assess the methodological quality and risk of bias of the studies. This evaluation was conducted independently by two reviewers (G.G.d.C.N. and F.C.A.d.M.), with disagreements resolved through discussion with a third reviewer (R.d.C.C.C.). The NOS examines three key domains: selection, comparability, and exposure, with maximum scores of 4, 2, and 3 stars, respectively, allowing a total possible score of 9. Additionally, the quality of reporting in the included studies was evaluated using the Strengthening the Reporting of Genetic Association (STREGA) guidelines [9]. These guidelines cover five major aspects: genotyping methods and errors, population stratification, haplotype variation, Hardy–Weinberg equilibrium, and replication. The first category includes five specific items, such as genotyping platform, error and call rates, batch processing, genotyping laboratories, and the number of successfully genotyped individuals. Each study was assessed across nine items, with one point assigned per item, resulting in a score range from 0 to 9, where higher scores reflect superior genetic study quality. This assessment was performed by three independent reviewers (G.G.d.C.N., F.C.A.d.M., and R.d.C.C.C.), with any disagreements resolved by the senior author (N.P.C.S.).

### 2.6. Data Analysis 

Statistical analyses were conducted using Review Manager (Rev Man), version 5.4.1 (The Cochrane Collaboration, Oxford, UK). Associations between the SNPs and GBM risk were calculated as a measure of pooled odds ratios (ORs) and 95% confidence intervals (CIs). We consider OR > 1 favoring increased risk of GBM and OR < 1 favoring decreased risk of GBM. Pooled OR was analyzed by the Mantel–Haenszel method (fixed-effect). The *I*^2^ range of 60–75% *I*^2^ was considered significant, indicating substantial heterogeneity. An *I*^2^ > 75% was considered to represent considerable heterogeneity.

### 2.7. Epidemiological Data Correlation

The incidence and mortality rates of brain and central nervous system cancers were obtained from the World Health Organization (WHO) platform, available through the Global Cancer Observatory [10]. Allele frequencies of genetic variants in continental populations were analyzed using data from the 1000 Genomes Project, specifically for Europe (EUR), Africa (AFR), East Asia (EAS), South Asia (SAS), and the Americas (AMR). The WHO population data were compared with the continental populations included in Phase 3 of the 1000 Genomes Project database (URL https://www.internationalgenome.org/, accessed on 4 October 2024). As per the Consortium of the 1000 Genomes Project, populations are grouped based on the predominant component of their ancestry. Consequently, our study correlated the epidemiological data from the WHO with the genetic data of the EUR, AFR, EAS, SAS, and AMR populations.

Pearson’s correlation was used for the brain, central nervous system cancer incidence, and allele frequencies of the variants. The data were evaluated with previously described groups, using the “cor. test” function of the “stats” package of the R programming language. Thereafter, and after passing the Bonferroni correction *p*-value threshold, the values of the r, r2, *p*-value, and 95% CI were obtained. All the plots were created using the “ggplot2” graphics package. It considered only *p*-value less than 0.05 (*p* ≤ 0.05) as statistically significant.

## 3. Results

### 3.1. Search Results 

**Figure 1:**
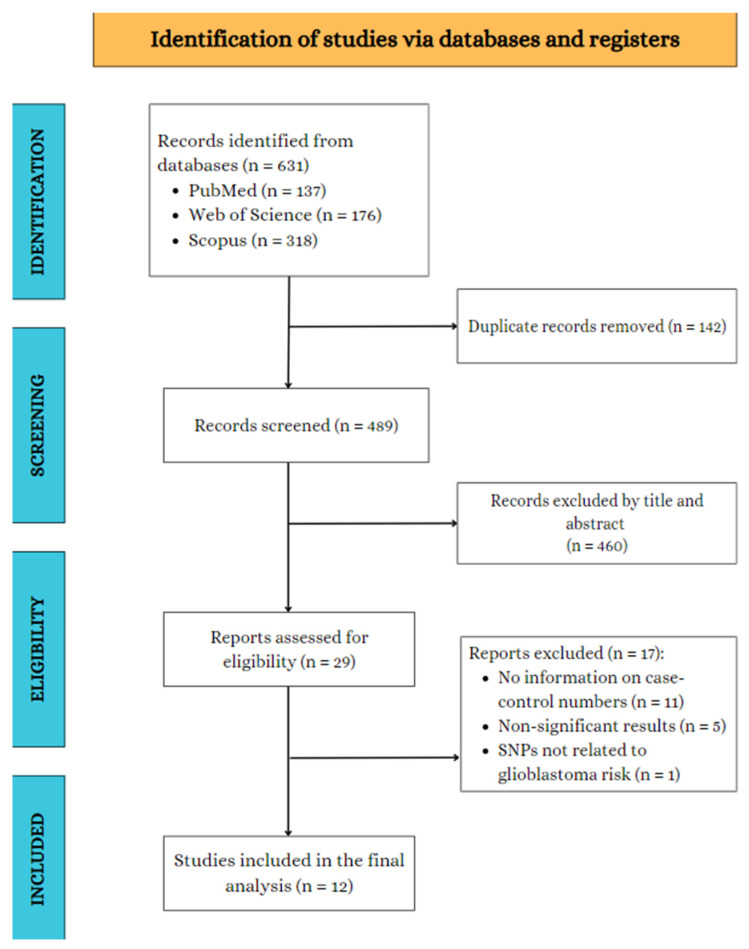
Study selection flowchart through literature search.

The electronic search identified 631 potentially relevant records. After removing duplicates, 489 records remained. Finally, after reviewing titles and abstracts, twelve articles were selected for full-text examination. Of these, only four met the inclusion criteria for the review (Jin Tian-Bo et al., 2013; Wei et al., 2014; Schwartzbaum et al., 2005; Li Bin et al., 2017) [11,12,13,14]. No relevant studies were identified from the reference lists of the included studies. Figure 1 presents a flowchart of the literature search process.

### 3.2. Characteristics of Studies, Genes/SNPs, and Participants 

The characteristics of the 12 studies are listed in Table 1. All the studies were published between 2005 and 2021. Data were collected from April 2020 to February 2022. In addition, the articles included populations from different countries; participants were from Europe (two studies), Asia (seven studies), North America (two studies), and Brazil (one study). The number of participants with GBM ranged from 72 to 1000. The predominant method used in studies for the genotyping was real-time reverse transcriptase-polymerase chain reaction. Total number of participants, number of GBM patients, gender, ethnicity, mean age and mean age at diagnosis, genotyping method, histologically confirmed GBM, and authors’ main finds were also shown in Table 1. A total of 22 SNPs related to 19 genes were collected, as shown in Table 2. Two SNPs were examined in two or more studies (*IL-4R* rs1801275 and *CCDC26* rs891835).

### 3.3. Quality Assessment

The methodological quality of the 12 studies, evaluated using the Newcastle–Ottawa Scale (NOS), is presented in Table 3. Total scores ranged from four to nine stars, with the lowest score of 7 awarded to studies by Jin Tian-Bo et al. (2013), Li Bin et al. (2017), and Al-Khatib et al. (2020) [14,21,22], and the highest score of 9 given to studies by Liu et al. (2014), Custódio et al. (2010), Jin Tianbo et al. (2013), and McKean-Cowdin et al. (2009) [11,16,17,19]. All studies received a star for items 1, 2, 3, and 4 in the “selection” domain, as well as for items 1 and 2 in the “exposure” domain. The “comparability” domain, which evaluates whether confounding factors between case and control groups were identified and adjusted in the analysis, allows a maximum of two stars. Five studies achieved the maximum score in this domain. The quality of reporting, assessed using the STREGA guidelines, is summarized in Table 4. Scores ranged from four to eight points, with McKean-Cowdin et al. (2009) and Mesic et al. (2021) [15,19] obtaining the highest scores, while Schwartzbaum et al. (2005) [13] received the lowest score.

### 3.4. Meta-Analysis

Only four of the twelve studies were selected for meta-analyses (Jin Tian-Bo et al., 2013; Wei et al., 2014; Schwartzbaum et al., 2005; Li Bin et al., 2017) [11,12,13,14].The studies by Mesic et al. (2021), Liu et al. (2014), Custódio et al. (2010), Dong et al. (2014), McKean-Cowdin et al. (2009), Rodriguez-Hernandez et al. (2013), Jin Tian-Bo et al. (2013), and Al-Khatib et al. (2020) [15,16,17,18,19,20,21,22] were not included because the investigated SNPs were not studied by other authors (Table 2).

**Figure 2:**
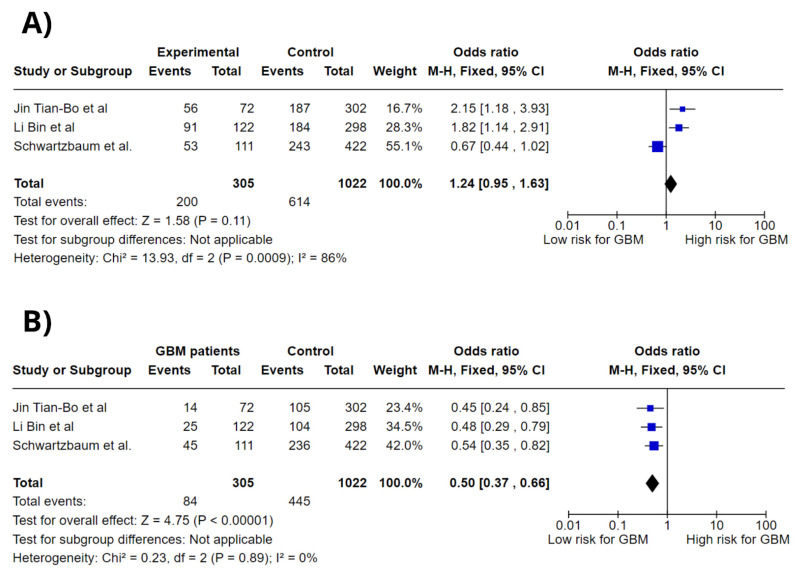
Forest plots of association between *IL-4R* gene polymorphisms and GBM risk. (**A**) *IL-4R* rs1801275 A/A codominant genotype. (**B**) *IL-4R* rs1801275 A/G codominant genotype [13,14,21].

Figure 2 shows forest plots of the association between *IL-4R* rs1801275 different genotypes and glioblastoma risk; the genotypes analyzed were A/A, A/G, and G/G, all in a codominant model. Meta-analysis showed an association between *IL-4R* rs1801275 A/A genotype and increased GBM risk (OR = 1.24; 95% CI: 0.95–1.63; *p* = 0.11; I^2^ = 0%). However, *IL-4R* rs1801275 A/G genotype was not associated with high GBM risk (OR = 0.50; 95% CI: 0.37–0.66; *p* < 0.00001; I^2^ = 0%). Overall, *IL-4R* rs1801275 showed no significant association with increased GBM risk.

**Figure 3:**
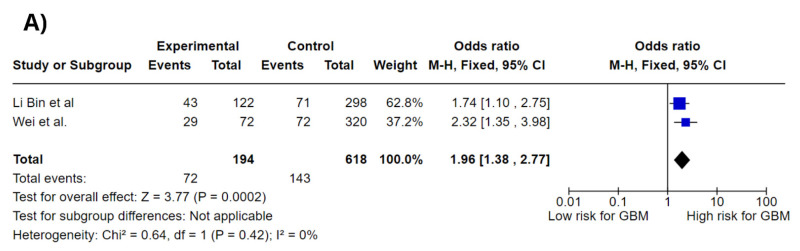

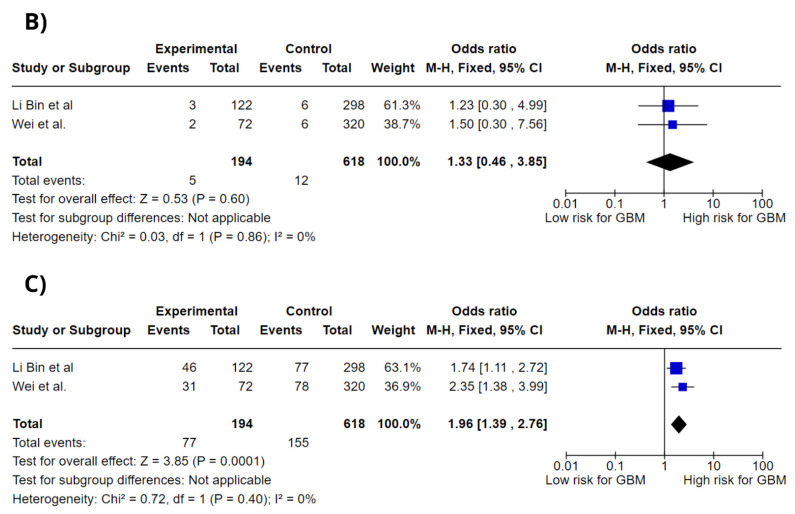
Forest plots of association between *CCDC26* gene polymorphisms and GBM risk. (**A**) *CCDC26* rs891835 G/T codominant genotype. (**B**) *CCDC26* rs891835 G/G codominant genotype. (**C**) *CCDC26* rs891835 G/T–G/G codominant genotype [12,14].

Figure 3 shows forest plots of the association between *CCDC26* rs891835 different genotypes and glioblastoma risk; the genotypes analyzed were G/T and G/G in a codominant model and G/T–G/G in a dominant model. Meta-analysis showed a significant association between all genotypes analyzed and increased GBM risk, *CCDC26* rs891835 G/T genotype (OR = 1.96; 95% CI: 1.38–2.77; *p* = 0.0002; I^2^ = 0%), G/G genotype (OR = 1.33; 95% CI: 0.46–3.85; *p* = 0.60; I^2^ = 0%), and G/T–G/G genotype (OR = 1.96; 95% CI: 1.39–2.76; *p* = 0.0001; I^2^ = 0%).

### 3.5. Epidemiological Correlation

**Figure 4:**
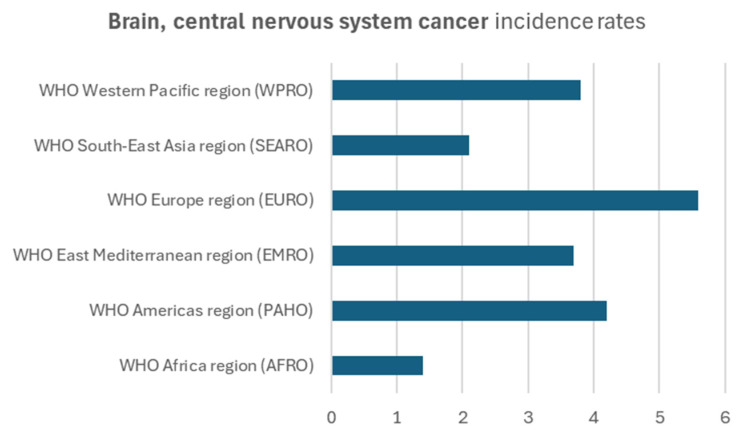
Brain, central nervous system cancer incidence rates per 100,000 habitants from the World Health Organization International Agency for Research on Cancer in different populations.

The Age-Standardized Incidence Rate (ASIR) for brain, central nervous system cancer indicates the frequency of new diagnoses of brain, central nervous system cancer within a defined timeframe, considering the distribution of age in the population. The incidence rate (ASIR) of brain, central nervous system cancer was 5.6 per 100,000, both sexes, in the European population, followed by the American (4.2), as shown in Figure 4.

**Figure 5:**
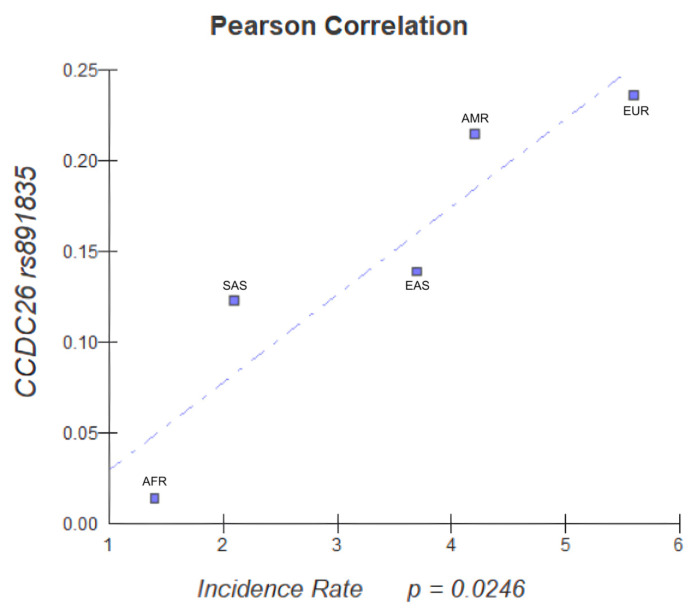
Pearson Correlation Analysis.

The variant *CCDC26* rs891835 was described as directly correlated in the Pearson Correlation Analysis (*p* value = 0.0246), as shown in Figure 5, and demonstrated that the higher the frequency of the variant, the higher the incidence of that variant in certain populations, especially in the European population. From this, we can observe an association between the frequency of the variant and higher incidence rates in the European population, a correlation that demonstrates that the variant rs891835, already identified in the meta-analysis as a relevant biomarker for glioblastoma risk, could serve as a screening marker in the European population.

## 4. Discussion

This is the first systematic review and meta-analysis to associate literature data with clinically significant variants and genes related to increased glioblastoma risk. It is also a pioneering study in correlating genetic variants with the global epidemiology of this brain tumor. Based on previous studies, we selected variants and genes potentially related to glioblastoma development.

Our analyses revealed a significant association between the *IL-4R* gene variant rs1801275 (A/A genotype) and glioblastoma susceptibility. Furthermore, the *CCDC26* gene variant rs891835 demonstrated an association with the disease. All genotypes of this variant (G/T, G/G, and G/T–G/G) were significantly associated with an increased risk of glioblastoma.

Studies have shown that the *IL-4* receptor-α (IL-4Rα) is overexpressed in glioma-infiltrating myeloid cells, playing a key role in driving their immunosuppressive functions. This overexpression is associated with the granulocyte-macrophage colony-stimulating factor (GM-CSF), which elevates IL-4Rα levels and enhances the activity of myeloid-derived suppressor cells (MDSCs), ultimately suppressing T-cell responses [23]. Studies show that this gene, along with the rs1801275 variant, is related to the likelihood of developing glioblastoma. Our results confirmed that the rs1801275 variant, particularly the A/G genotype, is strongly associated with an increased risk of glioblastoma in a superdominant model [21]. These data support the association of rs1801275 with glioblastoma susceptibility. However, in our analyses, only the A/A genotype of the *IL-4R* gene was associated with glioblastoma development.

The *CCDC26* gene, located on chromosome 8q24, has emerged as a significant player in various cancers, particularly gliomas and acute myeloid leukemia (AML). Research indicates that structural variations and polymorphisms in *CCDC26* are associated with tumorigenesis, influencing both the expression of oncogenes and patient prognosis [24]. Additionally, structural variations in *CCDC26* have been linked to pediatric gliomas, such as diffuse intrinsic pontine glioma (DIPG), where they contribute to elevated gene expression and tumor proliferation [25]. Studies show that the rs891835 variant in this gene increases glioblastoma susceptibility [12,14,26]. These findings are consistent with our analyses, which also identified a significant association between the rs891835 SNP in the *CCDC26* gene and increased glioblastoma risk, with higher frequency and incidence of this variant observed in populations of European descent.

## 5. Conclusions

The present study suggests that *CCDC26* rs891835 may serve as a predictive biomarker for GBM risk and may influence higher GBM incidence rates in the European population. While this study provides valuable insights, some aspects warrant consideration. The focus on previously reported SNPs could overlook unexplored genetic variants, and population heterogeneity might influence the generalizability of the findings. Future research could address these aspects by conducting larger longitudinal studies, exploring additional genetic variants and SNP–environment interactions, and investigating the molecular mechanisms underlying the role of these SNPs in glioblastoma pathogenesis.

## Figures and Tables

**Table 1:** Characteristics of included studies and participants.

Authors, Year	Country	Participants (Male/Female)	Mean Age	Mean Age at Diagnosis (Years)	Genotyping Method	GBM Histologically Confirmed	Ethnicity	Main Results
Mesic et al., 2021 [15]	Bosnia and Herzegovina	129 (68/61)	50 (19–81)	58	castPCR	Yes	European	rs2289590 in AURKB and rs11084490 in AURKC were associated with a reduced GBM risk
Liu et al., 2014 [16]	China	115 (58.5%/41.5%)	45.3	42.9	PCR	Yes	Chinese	*STAT5b* rs2293157 G/T was associated with increased GBM risk
Custódio et al., 2010 [17]	Brazil	80 (52/28)	45	NR	PCR-RFLP	NR	Brazilian	*GSTP1* rs947894 polymorphism was associated with increased GBM risk
Dong et al., 2014 [18]	China	72 (28/44)	44 ± 15	NR	locus-specific PCR	Yes	Chinese	Gene polymorphisms in *CHEK2*, *GSTP1*, and ERCC1 may be involved in GBM in the Han Chinese population
Jin Tianbo et al., 2013 [11]	China	72 (28/44)	NR	44 ± 15	locus-specific PCR	Yes	Chinese	Polymorphisms within *FLT3*, *EGFR*, NEIL3, and ALOX5 may contribute to the occurrence of GBM in the Han Chinese population
Wei et al., 2014 [12]	China	72 (28/44)	44 ± 15	41 ± 18	locus-specific PCR	Yes	Chinese	Genetic contribution of *CCDC26* to GBM progression among Han Chinese
McKean-Cowdin et al., 2009 [19]	United States of America	1015 (619/396)	56.3 ± 12.6	56	castPCR	Yes	North American	The C allele of the *PARP1* rs1136410 variant was associated with decreased GBM risk, G allele of the PRKDC rs7003908 was associated with increased GBM risk
Schwartzbaum et al., 2005 [13]	United States of America	111 (59.18% male)	56	65	DASH PCR	NR	North American	IL-4RA rs1805015 TC, CC and IL-4RA rs1801275 AG, AA were positively associated with GBM while IL-13 rs1800925 CT, TT was negatively associated with GBM
Rodriguez-Hernandez et al., 2013 [20]	Spain	115 (59.1% male)	63.2 ± 10.1	NR	castPCR	NR	European	*MLH1* rs1800734 and ERCC2 rs13181 polymorphisms might constitute glioblastoma susceptibility factors
Jin Tian-Bo et al., 2013 [21]	China	72 (28/44)	NR	44 ± 15	locus-specific PCR	Yes	Chinese	rs12645561 in NEIL3 and rs2291427 in ALOX5 were associated with increased GBM risk
Li Bin et al., 2017 [14]	China	122 (70/52)	46.89	NR	locus-specific PCR	NR	Chinese	*NFIL3*, *XRCC5*, *CCDC26*, TP53 and *IL-4R* genes associated with the risk of glioblastoma
Al-Khatib et al., 2020 [22]	Jordan	84 (60% male)	45.4	NR	locus-specific PCR	Yes	Arabic	*BRCA1* SNPs rs1799966 and rs799917 are associated with the risk of developing GBM in the Arab Jordanian population

NR: Not Reported.

**Table 2:** Investigated genes and polymorphisms.

Gene	SNPs	Study 1	2	3	4	5	6	7	8	9	10	11	12	Total
*IL-4R*	rs1801275					X			X			X		3
*IL-4R*	rs1805015										X			1
*CCDC26*	rs891835						X					X		2
*CCDC26*	rs6470745						X							1
*GSTP1*	rs1695				X									1
*GSTP1*	rs947894			X										1
*AURKB*	rs2289590	X												1
*AURKC*	rs11084490	X												1
*STAT5b*	rs2293157		X											1
*CHEK2*	rs2267130				X									1
*IL-3*	rs20541					X								1
*IL-10*	rs1800871					X								1
*PARP1*	rs1136410							X						1
*PRKDC*	rs7003908							X						1
*MLH1*	rs1800734									X				1
*ERCC2*	rs13181									X				1
*FLT3*	rs3829382										X			1
*XRCC5*	rs9288516											X		1
*NFIL3*	rs7021746											X		1
*TP53*	rs1042522											X		1
*BRCA1*	rs799917												X	1
*EGFR*	rs9642393										X			1

Study 1: Mesic et al., 2021; Study 2: Liu et al., 2014; Study 3: Custódio et al., 2010; Study 4: Dong et al., 2014; Study 5: Jin Tianbo et al., 2013; Study 6: Wei et al., 2014; Study 7: McKean-Cowdin et al., 2009; Study 8: Schwartzbaum et al., 2005; Study 9: Rodriguez-Hernandez et al., 2013; Study 10: Jin Tian-Bo et al., 2013; Study 11: Li Bin et al., 2017; Study 12: Al-Khatib et al., 2020 [11,12,13,14,15,16,17,18,19,20,21,22].

**Table 3:** Methodological quality of the studies based on the Newcastle–Ottawa Scale (NOS).

Studies	Selection	Item 2	Item 3	Item 4	Comparability	Item 1b	Exposure	Item 2	Item 3	Total Score
	Item 1				Item 1a		Item 1			
Mesic et al., 2021 [15]	*	*	*	*	*		*	*	*	8
Liu et al., 2014 [16]	*	*	*	*	*	*	*	*	*	9
Custódio et al., 2010 [17]	*	*	*	*	*	*	*	*	*	9
Dong et al., 2014 [18]	*	*	*	*	*	*	*	*		8
Jin Tianbo et al., 2013 [11]	*	*	*	*	*	*	*	*	*	9
Wei et al., 2014 [12]	*	*	*	*	*		*	*	*	8
McKean-Cowdin et al., 2009 [19]	*	*	*	*	*	*	*	*	*	9
Schwartzbaum et al., 2005 [13]	*	*	*	*	*		*	*	*	8
Rodriguez-Hernandez et al., 2013 [20]	*	*	*	*	*		*	*	*	8
Jin Tian-Bo et al., 2013 [21]	*	*	*	*	*		*	*		7
Li Bin et al., 2017 [14]	*	*	*	*	*		*	*		7
Al-Khatib et al., 2020 [22]	*	*	*	*	*		*	*		7

* Selection—Item 1: Is the case definition adequate?; Item 2: Representativeness of the cases; Item 3: Selection of controls; Item 4: Definition of controls. Comparability—Item 1a and 1b: Comparability of cases and controls based on the design or analysis. Exposure—Item 1: Ascertainment of exposure; Item 2: Same method of ascertainment for cases and controls; Item 3: Non-response rate.

**Table 4:** The quality of reporting using the Strengthening the Reporting of Genetic Association (STREGA) guideline.

Studies	Description of Genotyping Methods and Errors					Description of Modeling Population Stratification	Description of Modeling Haplotype Variation	Hardy-Weinberg Equilibrium Was Considered	Statement of Whether the Study is the First Report of a Genetic Association a Replication Effort, or Both	Score
	Genotyping Methods and Platforms	Error Rates and Call Rates	Genotyping in Batches	Laboratory/Center Where the Genotyping Was Done	The Numbers of Individuals Was Successful Genotyping					
Mesic et al., 2021 [15]	Yes	Yes	No	Yes	Yes	Yes	Yes	Yes	Yes	8
Liu et al., 2014 [16]	Yes	Yes	No	Yes	Yes	Yes	No	Yes	Yes	7
Custódio et al., 2010 [17]	Yes	No	No	Yes	Yes	Yes	No	Yes	Yes	6
Dong et al., 2014 [18]	Yes	No	No	Yes	Yes	Yes	No	Yes	Yes	6
Jin Tianbo et al., 2013 [11]	Yes	Yes	No	Yes	Yes	Yes	No	Yes	Yes	7
Wei et al., 2014 [12]	Yes	No	No	Yes	Yes	Yes	Yes	Yes	Yes	7
McKean-Cowdin et al., 2009 [19]	Yes	Yes	No	Yes	Yes	Yes	Yes	Yes	Yes	8
Schwartzbaum et al., 2005 [13]	Yes	Yes	No	Yes	Yes	No	No	No	No	4
Rodriguez-Hernandez et al., 2013 [20]	Yes	No	No	Yes	Yes	Yes	Yes	Yes	Yes	7
Jin Tian-Bo et al., 2013 [21]	Yes	Yes	No	Yes	Yes	No	No	Yes	Yes	6
Li Bin et al., 2017 [14]	Yes	No	No	Yes	Yes	Yes	No	Yes	Yes	6
Al-Khatib et al., 2020 [22]	Yes	No	No	Yes	No	No	No	Yes	Yes	4

## Data Availability

No new data were created or analyzed in this study. Data sharing is not applicable to this article.

## Supplementary Materials

The following supporting information can be downloaded at: https://www.mdpi.com/article/10.3390/jpm15090401/s1, Table S1: Single-nucleotide Polymorphisms (SNPs) selected from the global literature; Table S2: Search strategies.
