# Supplementary material for: Single-Nucleotide Polymorphisms Related to Glioblastoma Risk and Worldwide Epidemiology: A Systematic Review and Meta-Analysis
Source: J Pers Med. 2025 Sep 1;15(9):401. doi: 10.3390/jpm15090401 (PMC12470902; doi:10.3390/jpm15090401)
Supplement: Supplementary file 1 [file jpm-15-00401-s001.zip › jpm-3320412-supplementary.pdf]

Table S1. Single-nucleotide Polymorphisms (SNPs) selected from the global literature

Authors, Year	SNP	Gene
Mesic et al., 2021	rs2289590	AURKB
	rs11084490	AURKC
Liu et al., 2014	rs2293157	STATb5
Custódio et al., 2010	rs947894	GSTP1
Dong et al., 2014	rs1695	GSTP1
	rs2267130	CHEK2
Jin Tianbo et al., 2013	rs20541	IL-13
	rs1800871	IL-10
	rs1801275	IL-4R
Wei et al., 2014	rs891835	CCDC26
	rs6470745	CCDC26
McKean-Cowdin et al., 2009	rs1136410	PARP1
	rs7003908	PRKDC
Schwartzbaum et al., 2005	rs1805015	IL-4R
	rs1801275	IL-4R
Rodriguez-Hernandez et al., 2013	rs1800734	MLH1
	rs13181	ERCC2
Jin Tian-Bo et al., 2013	rs3829382	FLT3
	rs9642393	EGFR
Li Bin et al., 2017	rs9288516	XRCC5
	rs7021746	NFIL3
	rs891835	CCDC26
	rs1042522	TP53
	rs1801275	IL-4R
Al-Khatib et al., 2020	rs799917	BRCA1

Table S2. Search strategies

Database	Search strategy
PubMed (n= 137)	(((((((((("Glioblastoma"[MeSH Terms]) OR ("Glioblastomas"[MeSH Terms])) OR ("Astrocytoma, Grade IV"[MeSH Terms])) OR ("Astrocytomas, Grade IV"[MeSH Terms])) OR ("Grade IV Astrocytoma"[MeSH Terms])) OR ("Grade IV Astrocytomas"[MeSH Terms])) OR ("Glioblastoma Multiforme"[MeSH Terms])) OR ("Giant Cell Glioblastoma"[MeSH Terms])) OR ("Giant Cell Glioblastomas"[MeSH Terms])) OR ("Glioblastoma, Giant Cell"[MeSH Terms])) OR ("Glioblastomas, Giant Cell"[MeSH Terms]) AND (((((((("Genetic Predisposition to Disease"[MeSH Terms]) OR ("Genetic Susceptibility")) OR ("Genetic Susceptibilities")) OR ("Susceptibilities, Genetic")) OR ("Susceptibility, Genetic")) OR ("Genetic Predisposition")) OR ("Genetic Predispositions")) OR ("Predispositions, Genetic")) OR ("Predisposition, Genetic") OR (((((("Polymorphism, Single Nucleotide"[MeSH Terms]) OR ("Nucleotide Polymorphism, Single")) OR ("Nucleotide Polymorphisms, Single")) OR ("Polymorphisms, Single Nucleotide")) OR ("Single Nucleotide Polymorphisms")) OR ("SNPs")) OR ("Single Nucleotide Polymorphism") AND ((("Risk")[MeSH Terms]) OR (("Risk"[Title/Abstract]))) OR (("Risks"[Title/Abstract]))) OR (("Relative Risk"[Title/Abstract]))) OR (("Relative Risks"[Title/Abstract]))) OR (("Risk, Relative"[Title/Abstract]))) OR (("Risks, Relative"[Title/Abstract]))) OR (("Mortality")[MeSH Terms])) OR (("Mortality"[Title/Abstract]))) OR (("Mortalities"[Title/Abstract]))) OR (("Mortality Rate"[Title/Abstract]))) OR (("Mortality Rates"[Title/Abstract]))) OR (("Rate, Mortality"[Title/Abstract]))) OR (("Death Rate"[Title/Abstract]))) OR (("Death Rates"[Title/Abstract]))) OR (("Rate, Death"[Title/Abstract]))) OR (("Case Fatality Rate"[Title/Abstract]))) OR (("Case Fatality Rates"[Title/Abstract]))) OR (("Rate, Case Fatality"[Title/Abstract]))) OR (("Rates, Case Fatality"[Title/Abstract]))) OR (("CFR Case Fatality Rate"[Title/Abstract]))) OR (("Crude Death Rate"[Title/Abstract]))) OR (("Crude Death Rates"[Title/Abstract]))) OR (("Death Rate, Crude"[Title/Abstract]))) OR (("Rate, Crude Death"[Title/Abstract]))) OR (("Crude Mortality Rate"[Title/Abstract]))) OR (("Crude Mortality Rates"[Title/Abstract]))) OR (("Mortality Rate, Crude"[Title/Abstract]))) OR (("Rate, Crude Mortality"[Title/Abstract]))) OR (("Decline, Mortality"[Title/Abstract]))) OR (("Mortality Declines"[Title/Abstract]))) OR (("Mortality Decline"[Title/Abstract]))) OR (("Mortality Determinants"[Title/Abstract]))) OR (("Determinants, Mortality"[Title/Abstract]))) OR (("Determinant, Mortality"[Title/Abstract]))) OR (("Mortality Determinant"[Title/Abstract]))) OR (("Mortality, Differential"[Title/Abstract]))) OR (("Differential Mortality"[Title/Abstract]))) OR (("Differential Mortalities"[Title/Abstract]))) OR (("Age-Specific Death Rate"[Title/Abstract]))) OR (("Age-Specific Death Rates"[Title/Abstract]))) OR (("Death Rate, Age-Specific"[Title/Abstract]))) OR (("Rate, Age-Specific Death"[Title/Abstract]))) OR (("Age Specific Death Rate"[Title/Abstract]))) OR (("Mortality, Excess"[Title/Abstract]))) OR (("Excess Mortality"[Title/Abstract]))) OR (("Excess Mortalities"[Title/Abstract])))
Web of Science (n= 176)	((((((((((TS=("Glioblastoma")) OR TS=("Glioblastomas")) OR TS=("Astrocytoma, Grade IV")) OR TS=("Astrocytomas, Grade IV")) OR TS=("Grade IV Astrocytoma")) OR TS=("Grade IV Astrocytomas")) OR TS=("Glioblastoma Multiforme")) OR TS=("Giant Cell

	Glioblastoma")) OR TS=("Giant Cell Glioblastomas")) OR TS=("Glioblastoma, Giant Cell")) OR TS=("Glioblastomas, Giant Cell") AND (((((((((((((((ALL=("Genetic Predisposition to Disease")) OR ALL=("Genetic Susceptibility")) AND ALL=("Genetic Susceptibilities")) OR ALL=("Susceptibilities, Genetic")) OR ALL=("Susceptibility, Genetic")) OR ALL=("Genetic Predisposition")) OR ALL=("Genetic Predispositions")) OR ALL=("Predispositions, Genetic")) OR ALL=("Predisposition, Genetic")) OR ALL=("Polymorphism, Single Nucleotide")) OR ALL=("Nucleotide Polymorphism, Single")) OR ALL=("Nucleotide Polymorphisms, Single")) OR ALL=("Polymorphisms, Single Nucleotide")) OR ALL=("Single Nucleotide Polymorphisms")) OR ALL=("SNPs")) OR ALL=("Single Nucleotide Polymorphism") AND ((ALL=("Risk")) OR ALL=("Risks")) OR ALL=("Relative Risk")) OR ALL=("Relative Risks")) OR ALL=("Risk, Relative")) OR ALL=("Risks, Relative")) OR ALL=("Mortality")) OR ALL=("Mortalities")) OR ALL=("Mortality Rate")) OR ALL=("Mortality Rates")) OR ALL=("Rate, Mortality")) OR ALL=("Death Rate")) OR ALL=("Death Rates")) OR ALL=("Rate, Death")) OR ALL=("Case Fatality Rate")) OR ALL=("Case Fatality Rates")) OR ALL=("Rate, Case Fatality")) OR ALL=("Rates, Case Fatality")) OR ALL=("CFR Case Fatality Rate")) OR ALL=("Crude Death Rate")) OR ALL=("Crude Death Rates")) OR ALL=("Death Rate, Crude")) OR ALL=("Rate, Crude Death")) OR ALL=("Crude Mortality Rate")) OR ALL=("Crude Mortality Rates")) OR ALL=("Mortality Rate, Crude")) OR ALL=("Rate, Crude Mortality")) OR ALL=("Decline, Mortality")) OR ALL=("Mortality Declines")) OR ALL=("Mortality Decline")) OR ALL=("Mortality Determinants")) OR ALL=("Determinants, Mortality")) OR ALL=("Determinant, Mortality")) OR ALL=("Mortality Determinant")) OR ALL=("Mortality, Differential")) OR ALL=("Differential Mortality")) OR ALL=("Differential Mortalities")) OR ALL=("Age-Specific Death Rate")) OR ALL=("Age-Specific Death Rates")) OR ALL=("Death Rate, Age-Specific")) OR ALL=("Rate, Age-Specific Death")) OR ALL=("Age Specific Death Rate")) OR ALL=("Mortality, Excess")) OR ALL=("Excess Mortality")) OR ALL=("Excess Mortalities")
Scopus (n= 318)	TITLE-ABS-KEY ("Glioblastoma") OR TITLE-ABS-KEY ("Glioblastomas") OR TITLE-ABS-KEY ("Astrocytoma, Grade IV") OR TITLE-ABS-KEY ("Astrocytomas, Grade IV") OR TITLE-ABS-KEY ("Grade IV Astrocytoma") OR TITLE-ABS-KEY ("Grade IV Astrocytomas") OR TITLE-ABS-KEY ("Glioblastoma Multiforme") OR TITLE-ABS-KEY ("Giant Cell Glioblastoma") OR TITLE-ABS-KEY ("Giant Cell Glioblastomas") OR TITLE-ABS-KEY ("Glioblastoma, Giant Cell") OR TITLE-ABS-KEY ("Glioblastomas, Giant Cell") AND TITLE-ABS-KEY ("Genetic Predisposition to Disease") OR TITLE-ABS-KEY ("Genetic Susceptibility") AND TITLE-ABS-KEY ("Genetic Susceptibilities") OR TITLE-ABS-KEY ("Susceptibilities, Genetic") OR TITLE-ABS-KEY ("Susceptibility, Genetic") OR TITLE-ABS-KEY ("Genetic Predisposition") OR TITLE-ABS-KEY ("Genetic Predispositions") OR TITLE-ABS-KEY ("Predispositions, Genetic") OR TITLE-ABS-KEY ("Predisposition, Genetic") OR TITLE-ABS-KEY ("Polymorphism, Single Nucleotide") OR TITLE-ABS-KEY ("Nucleotide Polymorphism, Single") OR TITLE-ABS-KEY ("Nucleotide Polymorphisms, Single") OR TITLE-ABS-KEY ("Polymorphisms, Single Nucleotide") OR TITLE-ABS-KEY ("Single Nucleotide Polymorphisms") OR TITLE-ABS-KEY ("SNPs") OR TITLE-ABS-KEY ("Single Nucleotide Polymorphism") AND TITLE-ABS-KEY ("Risk") OR TITLE-ABS-KEY ("Risks") OR TITLE-ABS-KEY ("Relative Risk") OR TITLE-ABS-KEY ("Relative Risks") OR TITLE-ABS-KEY ("Risk, Relative") OR TITLE-ABS-KEY ("Risks, Relative") OR TITLE-ABS-KEY ("Mortality") OR TITLE-ABS-KEY ("Mortalities") OR TITLE-ABS-KEY ("Mortality Rate") OR TITLE-ABS-KEY ("Mortality Rates") OR TITLE-ABS-KEY ("Rate, Mortality") OR TITLE-ABS-KEY ("Death Rate") OR TITLE-ABS-KEY ("Death Rates") OR TITLE-ABS-KEY ("Rate, Death") OR TITLE-ABS-KEY ("Case Fatality Rate") OR TITLE-ABS-KEY ("Case Fatality Rates") OR TITLE-ABS-KEY ("Rate, Case Fatality") OR TITLE-ABS-KEY ("Rates, Case Fatality") OR TITLE-ABS-KEY ("CFR Case Fatality Rate") OR TITLE-ABS-KEY ("Crude Death Rate") OR TITLE-ABS-KEY ("Crude Death Rates") OR TITLE-ABS-KEY ("Death Rate, Crude") OR TITLE-ABS-KEY ("Rate, Crude Death") OR TITLE-ABS-KEY ("Crude Mortality Rate") OR TITLE-ABS-KEY ("Crude Mortality Rates") OR TITLE-ABS-KEY ("Mortality Rate, Crude") OR TITLE-ABS-KEY ("Rate, Crude Mortality") OR TITLE-ABS-KEY ("Decline, Mortality") OR TITLE-ABS-KEY ("Mortality Declines") OR TITLE-ABS-KEY ("Mortality Decline") OR TITLE-ABS-KEY ("Mortality Determinants") OR TITLE-ABS-KEY ("Determinants, Mortality") OR TITLE-ABS-KEY ("Determinant, Mortality") OR TITLE-ABS-KEY ("Mortality Determinant") OR TITLE-ABS-KEY ("Mortality, Differential") OR TITLE-ABS-KEY ("Differential Mortality") OR TITLE-ABS-KEY ("Differential Mortalities") OR TITLE-ABS-KEY ("Age-Specific Death Rate") OR TITLE-ABS-KEY ("Age-Specific Death Rates") OR TITLE-ABS-KEY ("Death Rate, Age-Specific") OR TITLE-ABS-KEY ("Rate, Age-Specific Death") OR TITLE-ABS-KEY ("Age Specific Death Rate") OR TITLE-ABS-KEY ("Mortality, Excess") OR TITLE-ABS-KEY ("Excess Mortality") OR TITLE-ABS-KEY ("Excess Mortalities")
